# Inbreeding affected differently on observations distribution of a growth trait in Iranian Baluchi sheep

**DOI:** 10.5713/ajas.19.0642

**Published:** 2020-02-25

**Authors:** Fateme Bahri Binabaj, Seyyed Homayoun Farhangfar, Majid Jafari

**Affiliations:** 1 Department of Animal Science, College of Agriculture and Natural Resources, Gonbad Kavous University, Gonbad Kavous, PO Box 163, Iran; 2 Department of Animal Science, College of Agriculture, University of Birjand, Birjand 9717434765, Iran; 3 Jihad Agricultural Organization, Mashhad, PO Box 91735-484, Iran

**Keywords:** Baluchi Sheep Breed, Non-genetic Factors, Growth Trait, Inbreeding, Quantile Regression

## Abstract

**Objective:**

Initial consequence of inbreeding is inbreeding depression which impairs the performance of growth, production, health, fertility and survival traits in different animal breeds and populations. The effect of inbreeding on economically important traits should be accurately estimated. The effect of inbreeding depression on growth traits in sheep has been reported in many breeds. Based on this, the main objective of the present research was to evaluate the impact of inbreeding on some growth traits of Iranian Baluchi sheep breed using quantile regression model.

**Methods:**

Pedigree and growth traits records of 13,633 Baluchi lambs born from year 1989 to 2016 were used in this research. The traits were birth weight, weaning weight, six-month weight, nine-month weight, and yearling weight. The contribution, inbreeding and co-ancestry software was used to calculate the pedigree statistics and inbreeding coefficients. To evaluate the impact of inbreeding on different quantiles of each growth trait, a series of quantile regression models were fitted using QUANTREG procedure of SAS software. Annual trend of inbreeding was also estimated fitting a simple linear regression of lamb’s inbreeding coefficient on the birth year.

**Results:**

Average inbreeding coefficient of the population was 1.63 percent. Annual increase rate of inbreeding of the flock was 0.11 percent (p<0.01). The results showed that the effect of inbreeding in different quantiles of growth traits is not similar. Also, inbreeding affected differently on growth traits, considering lambs’ sex and type of birth.

**Conclusion:**

Quantile regression revealed that inbreeding did not have similar effect on different quantiles of growth traits in Iranian Baluchi lambs indicating that at a given age and inbreeding coefficient, lambs with different sex and birth type were not equally influenced by inbreeding.

## INTRODUCTION

Mutton makes up the most important source of red meat in Iran. Small ruminants, especially sheep, play a key role in enhancing the husbandry economy of this country, although they have failed to satisfy the growing consumer demand. Promoting the capacity of breeding lambs is a sustainable alternative to increase meat production and improve the breeding efficiency in any sheep breeding enterprise [1].

The fat-tailed and small-sized Baluchi is an important Iranian sheep breed that accounts for slightly over 12 percent of the total sheep population in Iran. Baluchi sheep can well adapt to dry and hot climatic conditions in eastern Iran, especially in Khorasan Razavi Province, and thanks to their large population, they play a major role in the total lamb and mutton production in Iran [2].

Inbreeding refers to the likelihood of two alleles at any lo cus being identical by descent [3]. Inbreeding mainly occurs for high intensities of selection while intensively using a few animals for breeding. A small number of the seedstock with significant familial relationships are therefore responsible for maintaining nearly the entire genetic pool in the population [4].

Although inbreeding used to be considered useful for improving livestock population through the frequency increase of the desired genotypes, it usually causes economic loss [5]. Inbreeding depression emerging as a change in performance per unit of inbreeding increase is generally known as an initial consequence of inbreeding. Inbreeding depression degrades the growth, production, health, reproduction and survival traits [6–9]. Different breeds, populations and traits apparently vary in terms of their responses to inbreeding [5,10]. The inbreeding rate and its effect on growth associated traits in sheep have been reported in many breeds along with a wide range of changes in the relevant estimations [4, 11,12]. Inbreeding depression was respectively reported as −0.001, −0.01, −0.018, −0.01 and −0.13 per 1% increase in inbreeding in Baluchi sheep for body weights upon birth, and at the age of 3, 6, 9, and 12 months [13].

Inbreeding is therefore an important parameter that should be monitored and controlled in breeding programs. The inbreeding rate should be limited to maintain diversity at an acceptable level and cause genetic variation to ensure that future animals can respond to environmental changes and to selection. Animals cannot adapt themselves to these changes in the absence of genetic variation [6]. The changes in economically significant traits related to increases in inbreeding should be accurately estimated [5]. Quantile regression is a new method for estimating these effects which provides an inbreeding coefficient for each quantile of the desired trait.

Our hypothesis was that the impact of inbreeding is not the same on observations distribution of a growth trait. Based on this, the main objective of the present research was to evaluate the impact of inbreeding on some growth traits of Iranian Baluchi sheep breed using quantile regression model.

## MATERIALS AND METHODS

### Flock management

The data and pedigree information used in the present study were collected in 1989 to 2016 from Animal Breeding Station in Khorasan Razavi province in north-east Iran. This station has selected the rams and ewes of the Baluchi breed based on their breed characteristics, weaning weight (WW), body conformation, wool quality score and birth type. The sheep in this herd are generally provided with shelter at night and free access to hay, water and mineral lick blocks and are routinely treated against internal and external parasites [14]. At the age of about 18 months, maiden ewes are randomly exposed for the first time to selected 3 to 4-year-old rams. Mating is controlled, and 20 to 25 ewes are allocated in single-sire pens and mated with one ram of the same breed. August to late October is the breeding season of sheep as a mono-estrous mammal, and lambing subsequently occurs in late January to March. The lambs are ear tagged at birth, and information about their sex, birth weight (BW) and birth type, dam and sire identification numbers are recorded in herd books as well. Body weight is also recorded at weaning and 6, 9, and 12 months of age. Lambs suckle their mothers and get access to creep fed with good quality alfalfa hay *ad libitum* from 30 days of age, and are weaned at an average age of 90±10 days. During spring and summer, the flock is allowed to graze on natural pasture, and, kept indoors in autumn and winter, and fed with a ratio, based upon wheat and barley stubble [2].

### Data and statistical analysis

The present study used the pedigree and weight traits records of 13,633 lambs as the descendants of 3,747 dams and 308 rams. The traits comprised BW, WW, six-month weight (6MW), nine-month weight (9MW), and yearling weight (YW). Primary data file was edited for doubtful and out of range observations. Table 1 presents the descriptive statistics of weight traits.

The contribution, inbreeding and co-ancestry (CFC) soft ware [15] was used to calculate the pedigree statistics for the whole population and inbreeding coefficients for each individual in the pedigree. A modified algorithm of Colleague [15] is used in the CFC to calculate inbreeding coefficients. Table 2 summarizes the pedigree structure of the data used in the present study. According to the inbreeding coefficients obtained from their pedigrees, all the animals were grouped into four categories. The first category included non-inbred animals (F = 0), while the second, third and fourth categories included inbred animals respectively with 0<F≤0.1, 0.1< F≤0.2, and F>0.2. Inbreeding coefficients were considered zero for the animals in the founder population given that their parents were unknown and their pedigree information were unavailable.

The relationship between the conditional mean of a re sponse variable and one or more independent covariates can be modeled by ordinary least-squares regression, but quantile regression first introduced by Koenker and Bassett [16], models the relationship between independent covariates and the conditional quantiles of response variable. This model is especially useful when extremes i.e. lower and upper quantiles are important and can give a more complete picture of the conditional distribution of response variable when quantiles or the extremes are of interest [17]. Also it is useful if the rate of changes in the conditional quantile, expressed by regression coefficients, is dependent on the quantile. The flexibility in modeling data with heterogeneous conditional distribution constitutes the main advantage of quantile regression compared with ordinary least-squares regression. This type of data can be collected in many fields, including ecology, survival analysis and econometrics [18]. When a set of percentiles is modeled, quantile regression can provide a comprehensive picture of covariate effect, while making no distributional assumptions about the error term in the model [17]. Therefore, as a pioneer study, the QUANTREG procedure of SAS software [19] was applied to fit a series of quantile regression models to the data in order to estimate the effects of inbreeding on different percentiles of each growth trait (model 1). In the context of ordinary least squares, the model is as follows:

(1) $$\begin{array}{c} {\text{y}}_{\text{ijklmn}}=\mu +{\text{by}}_{\text{i}}+{\text{bm}}_{\text{j}}+{\text{sex}}_{\text{k}}+{\text{bt}}_{1}+{\text{dage}}_{\text{m}}+{\left(\text{sex}\times \text{bt}\right)}_{\text{kl}}+{\left(\text{sex}\times \text{dage}\right)}_{\text{km}}\\ +{\left(\text{bt}\times \text{dage}\right)}_{\text{lm}}+{\text{b}}_{1}({\text{bw}}_{\text{ijklmn}}-\bar{\text{bw}})+{\text{b}}_{2}({\text{F}}_{\text{ijklmn}}-\bar{\text{F}})+{\text{e}}_{\text{ijklmn}} \end{array}$$

In the context of quantile regression, the model can be written as:

(2) $$E(y|X=x)=x^{\prime }\beta$$

For solving:

(3) $$\hat{\beta }=\text{arg }{\text{min}}_{\beta \in R^{P}}\sum_{i}^{n}{\left(y_{i}-{x^{\prime }}_{i}\beta \right)}^{2}$$

The linear conditional quantile function; *Q*(*τ*|*X* = *x*) = *x*′ *β*(*τ*) can be estimated by solving:

(4) $$\hat{\beta }(\tau )=\text{arg }{\text{min}}_{\beta \in R^{P}}\sum_{i}^{n}\rho_{\tau }(y_{i}-{x^{\prime }}_{i}\beta )$$

for any quantile *τ* ∈ (0,1) and *β̂*(*τ*) the quantity is called the *τ*th regression quantile.

In the first model, y _ijklmn_ the observation of growth trait; μ the overall mean (intercept of the model); by_i_ the year of birth (i = 1989 to 2016); bm_j_ the month of birth (j = Jan, Feb, and Mar); sex_k_ the sex of lamb (k = male or female); bt_i_ the type of birth (l = single or twin); dage_m_ the age of dam at lambing (m = 2 to 7 years old); b_1_ regression coefficient of BW, bw_ijklmn_ the co-variable effect of BW (included in the model for all traits except BW); b_2_ regression coefficient of inbreeding, F_ijklmn_ the co-variable effect of inbreeding coefficient of the individual; e_ijklmn_ the random residual effect.

Annual trend of inbreeding was also estimated fitting a simple linear regression of lamb’s inbreeding coefficient on the birth year.

## RESULTS

The inbreeding coefficients estimated for this population was 0% to 31.25% with a mean of 1.63%, which was lower than that obtained in the Moghani (2.93%) and Baluchi (1.79%) sheep in Iran and in the Santa Ines sheep in Brazil (2.33%) [4,13,20]. The inbreeding coefficients estimated in other Iranian sheep breeds such as Lori (0.69%) and Guilan (0.15%) were below that in the present research [12,21]. The present estimates are consistent with previously-published reports [2,22]. Almost 6,047 heads accounting for 44.35% of the entire population were inbred with an average inbreeding coefficient of 3.68%, which was higher than that of Moghani (2.062%) and lower than Shal (6.28%) breeds [22,23]. Inbreeding is a special parameter in each population, and differences in inbreeding estimates among populations can be attributed to the variations in the accuracy of data recording, pedigree depth and completeness, mating programs, genetic selection schemes, genetic structure of the population and management. High inbreeding rates in some populations can be associated with the low number of their base population [5].

Table 3 presents the frequency of animals by the category of inbreeding coefficient. The pedigree analysis revealed that only 78 (0.58%) out of 13,633 animals were highly inbred (F>0.2), potentially due to sires’ mating with their (grand) daughters and extensively using a few rams in the herd as sires. Increasing the number of breeding males and their frequent replacement with new proved rams can therefore help reduce inbreeding levels. The non-inbred group (F = 0) included approximately 56% of the animals. In line with the present findings, the highest inbreeding class was reported to include the minimum number of animals, and the non-inbred class the maximum number [21–23].

**Figure 1:**
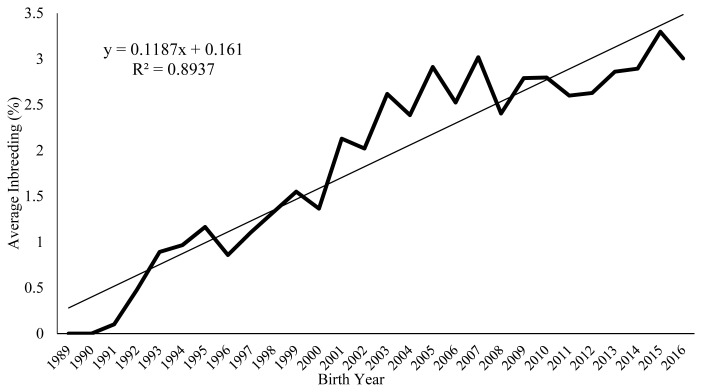
Inbreeding trend in Baluchi herd over the years.

Figure 1 shows the changes in the mean inbreeding rates between 1989 and 2016, suggesting that in addition to its fluctuation observed in different years, the inbreeding rate of this herd has progressively increased with an annual increase of 0.11% (p<0.01). The average inbreeding coefficient obtained in this flock was higher than that obtained for Moghani (0.02%) [13] and Shal sheep (0.07%) [22], and lower than Lori (0.21%) [21] and Elsenburg Dormer sheep (1.53%) [6]. This alarmingly increasing trend suggests the need for developing a mating plan in this flock to avoid further mating of close relatives.

### Effect of inbreeding on different quantiles of birth weight

A decrease was observed in BW by 0 to 5.3 g with a 1% increase in inbreeding rates, which is consistent with many studies. Ghavi Hossein-Zadeh [13], Mokhtari et al [24], and Sheikhloo et al [25] reported a regression coefficient of −9.5 g in the Moghani sheep breed, −7 g in the Iran Black breed and −7 g in the Baluchi sheep breed. Selvaggi et al [26] reported a 19 g reduction in BW in the Leccess sheep and Pedrosa et al [4] a 3.4 g reduction in the Santa Ines sheep with every 1% increase in inbreeding. These different effects of inbreeding can be explained by the differences between the breeds in terms of allele segregation, genetic variations in the base population, management and diversity of the founders of the flocks examined [27].

**Figure 2:**
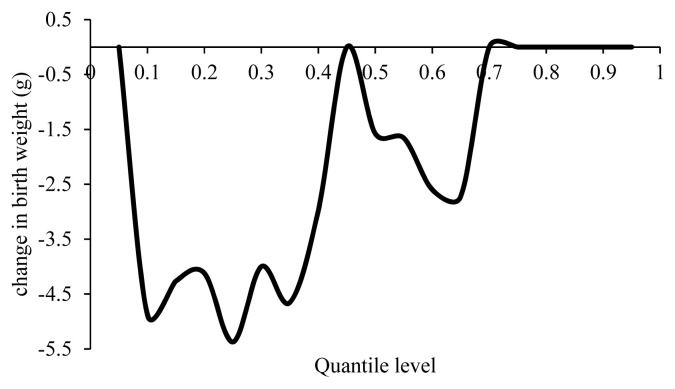
Estimated effect of inbreeding on different quantiles of birth weight.

According to Figure 2, the effect of inbreeding depression on lambs with a BW below the average (4.2 kg) was higher than on those with a BW over the average. The BW remained unchanged in the 45th percentile, while it decreased with an increase in inbreeding in the 45th to 65th percentiles. The effect of inbreeding depression on BW decreased in the 75th to 100th percentile.

**Figure 3:**
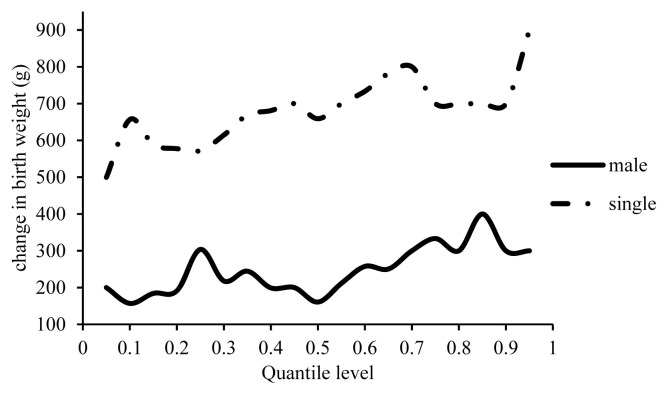
Estimated effect of inbreeding on different quantiles of birth weight in male and single lambs.

The different effects of inbreeding on the body weight of male and female lambs of the same age can be explained by physiological, genetic and hormonal differences [12,13,21]. Figure 3 shows an increase in the BW of male lambs by 160 to 400 g compared to females with a 1% increase in inbreeding. This increase was higher in males whose BWs were higher than the herd’s average. With a 1% increase in inbreeding, the increase in the BW of the male lambs was higher than that of females by 160 g at the 50th percentile as the midpoint. In line with the present study, Ghavi Hossein-Zadeh [13] reported 9 g increase in the BW of male Moghani lambs with a 1% increase in inbreeding, whereas, a 7 g increase was reported in the BW of female Lori lambs [21]. Eteqadi et al [12] reported a decrease in the BW of both male and female lambs with a 1% increase in inbreeding. Inbreeding depression can be further reduced in males by sex chromosome homozygosity, which affects the dominance of certain mutations, and by the absence of sex-based selection [28].

The BW of the single lambs was higher than that of twin lambs by 500 to 900 g with a 1% increase in inbreeding (Figure 3). A reduction by 5.6 g was reported in the BW of single Guilan lambs, and by 2.16 g in the twin ones [12]. This reduction was 9 g in the single Moghani lambs and 12 g in the twin ones [13]. In contrast, an insignificant increase of 0.5 g was reported in the BW of single lambs in the Lori sheep breed [21]. The difference in the BW of single and twin lambs can be explained by the differences in the uterus condition of pregnant dams. Multiple pregnancies negatively affect capillaries feeding a fetus, as each fetus receives fewer nutrients compared to in a single pregnancy, causing a lower BW.

### Effect of inbreeding on different quantiles of weaning weight

**Figure 4:**
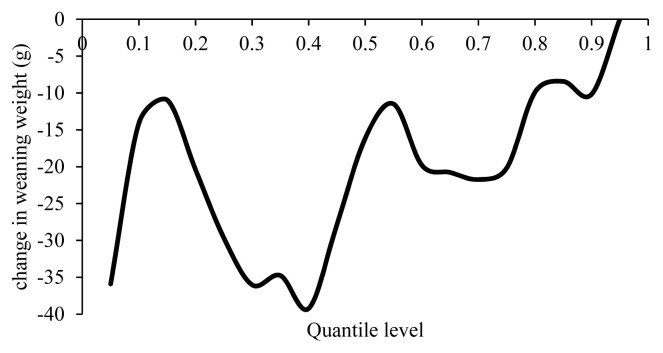
Estimated effect of inbreeding on different quantiles of weaning weight.

The rate of changes in quantile regression coefficients of WW with 1% increase in lamb’s inbreeding were −40 to 0 g (Figure 4). Eteqadi et al [12] reported a 28.4 g reduction in WW with a 1% increase in inbreeding, Yavarifard et al [8] a 14.68 g reduction, Van Wyk et al [6] a 9 g reduction and Negussie et al [10] 6 g reduction, which are consistent with the present research. The estimates of inbreeding depression in the WW of the Baluchi sheep (−87 g), Moghani sheep (−291 g), and Muzaffarnagari sheep (−48 g) were higher in magnitude than those of the present study [11,23,25]. In contrast, Ghavi Hossein-Zadeh [13] reported a 57 g increase in the WW of the Moghani sheep breed with a 1% increase in inbreeding.

Figure 4 shows that the maximum inbreeding depression of −40 g at this age occurs in the 40th percentile of WW, although a decline of 16 g is observed in WW in the mean point i.e. WW of 21.67 g with a 1% increase in inbreeding. Lambs weaned at weights below the average were therefore more susceptible to inbreeding depression.

**Figure 5:**
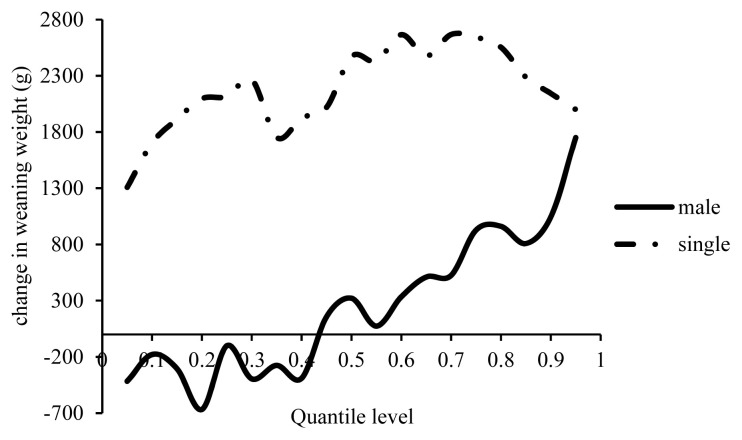
Estimated effect of inbreeding on different quantiles of weaning weight in male and single lambs.

In terms of sex, the weight of male lambs was lower than the females by 100 to 660 g with a 1% increase in inbreeding form the first to the 40th percentile of WW, although the WW of the male lambs increased with a 1% increase in inbreeding from the 45th to the 100th percentile, in which they were heavier than the females by 1,750 g (Figure 5). In other words, inbreeding positively affected the males weaned at higher weights compared to females. To the best of the authors’ knowledge, the results have not yet been compared in literature, although the effect of inbreeding has been separately reported on male and female lambs. Eteqadi et al [12] and Yeganehpur et al [21] estimated a 30 g decrease in the WW of male and 26 g in female Guilan lambs with a 1% increase in inbreeding, and a 12 g reduction in male and 11 g in female Lori sheep lambs. Increasing inbreeding by 1% increased WW in male Moghani lambs by 126 g which is consistent with the present results [13]. According to Figure 5, WW was higher in single-born lambs 1,300 to 2,700 g compared to in twin lambs after inbreeding increased by 1%, especially in WWs over the average. Increasing the inbreeding level decreased WW by 27 g in single-born and by 75 g in twin Guilan lambs, and by 16 g in single-born and 1.7 g in twin Lori lambs [12,21]. Insignificant increases were estimated in the WW of single-born Moghani lambs by 7 g and in the twins by 129 g [13]. The main factors affecting the diversity of the effects of inbreeding include levels of genetic variation in the base population, differences between the breeds in allele segregation and diversity of the founders in the tested flocks [27].

### Effect of inbreeding on different quantiles of 6-month weight

**Figure 6:**
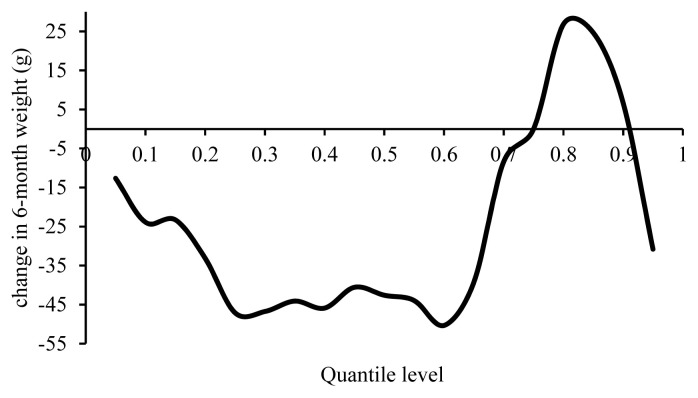
Estimated effect of inbreeding on different quantiles of 6-month weight.

Figure 6 suggests dissimilarities between the effects of a 1% increase in inbreeding on different percentiles of 6MW, ranging from a 50 g decrease to a 26 g increase. Only a decrease was reported in 6MW of certain breeds [4,21,22], whereas an increase was reported in some others [2,12,21]. According to Figure 6, a 1% change in inbreeding reduced 6MW from the 1st to 60th percentiles, although the reducing effect of inbreeding diminished from the 60th percentile onwards. Overall, inbreeding depression was small or even increased the animals’ weight at percentiles over the average 6MW.

**Figure 7:**
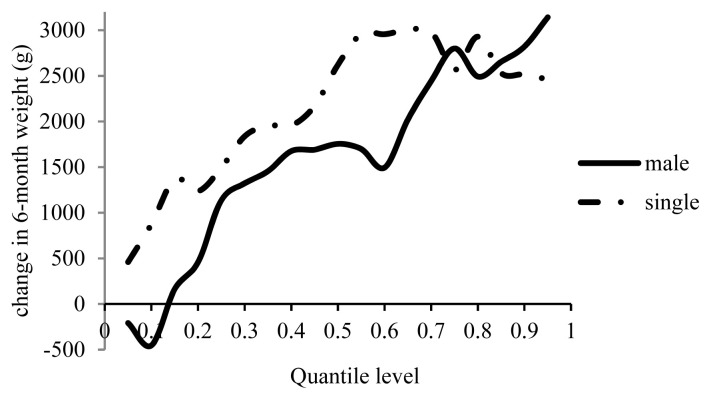
Estimated effect of inbreeding on different quantiles of 6-month weight in male and single lambs.

Figure 7 shows that 6MW was lower in the male lambs than in the females in the first two percentiles, while the males were heavier than the females by 162 to 3,100 g with a 1% increase in inbreeding from the 10th to the 100th percentile. Six-month weight increased by 17 g in male Guilan lambs and by 81 g in male Moghani lambs with a 1% increase in inbreeding [12,13]. An inbreeding depression of −83 g was observed in female Lori lambs [21] and −234 g in female Moghani sheep [13], whereas the 6MW of female lambs increased by 22 g in the Guilan breed with a 1% increase in inbreeding [12]. At the same inbreeding level, 6MW was higher in single-born lambs than in twin lambs (Figure 7). Increasing inbreeding by 1% caused the 6MW of the singletons to exceed that of the twins by 460 to 3,000 g. Given the different types of birth, inbreeding has reportedly affected 6MW differently. The 6MW of the singleton lambs increased by 20 g in the Guilan breed and by 29 g in the Moghani breed with a 1% increase in inbreeding, although it decreased by 95 g in the Lori breed. Increasing inbreeding by 1%, however, respectively reduced 6MW in the twins of the same breeds by 413, 8.1, and 128 g [12,13,21].

### Effect of inbreeding on different quantiles of 9-month weight

**Figure 8:**
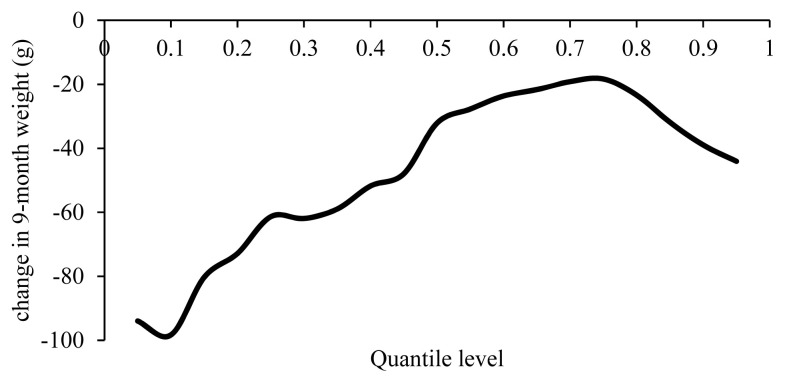
Estimated effect of inbreeding on different quantiles of 9-month weight.

A 1% increase in inbreeding reduced 9MW by 20 to 100 g (Figure 8). The inbreeding depression obtained in the present study was consistent with the estimates of −77 g in the Lori sheep and −45 g in the Moghani sheep [13,21]. Lower inbreeding depressions reported included a 10 g reduction in the 9MW of the Baluchi sheep, 19 g in the Shal, 19 g in the Moghani and 13 g in the Hissardale [2,22,23,29]. A 1% increase in inbreeding caused a more significant reduction in 9MW in percentiles below the mean. Inbreeding depression was obtained as −32 g at the average 9MW weight (i.e 33.6 g), whereas the lowest inbreeding effect on 9MW was observed at the 70th and 75th percentiles.

**Figure 9:**
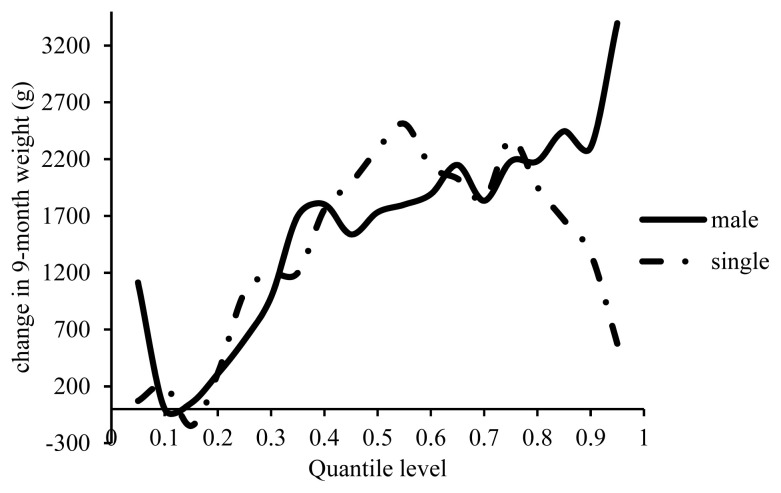
Estimated effect of inbreeding on different quantiles of 9-month weight in male and single lambs.

Male lambs were heavier than females by 50 to 3,300 g over the entire range of the percentiles of 9MW (Figure 9); the higher the percentile, the heavier the male lambs. The insignificant effects of inbreeding depression on 9MW were reported as 114 g in male Lori lambs and −71 g in male Moghani lambs [13,21]. With a 1% increase in inbreeding 9MW decreased by 233 g in the female Lori lambs and by −25 g in the female Moghani lambs. According to the present findings, increasing inbreeding by 1% caused the weight of single-born lambs to range from 147 g lighter to 2,500 g heavier compared to that of twin lambs over the entire range of percentiles (Figure 9). Type of birth has rarely been addressed in studies investigating the effect of inbreeding on 9MW. The inbreeding depression of 9MW has been reported as −109 g and 3 g in single-tone lambs of Lori and Moghani sheep breeds, respectively [13,21]. A 1% increase in inbreeding caused insignificant changes in 9MW, including an increase by 145 g in the twin Lori breed and a decrease by 166 g in the twin Moghani breed [13,21].

### Effect of inbreeding on different quantiles of yearling weight

**Figure 10:**
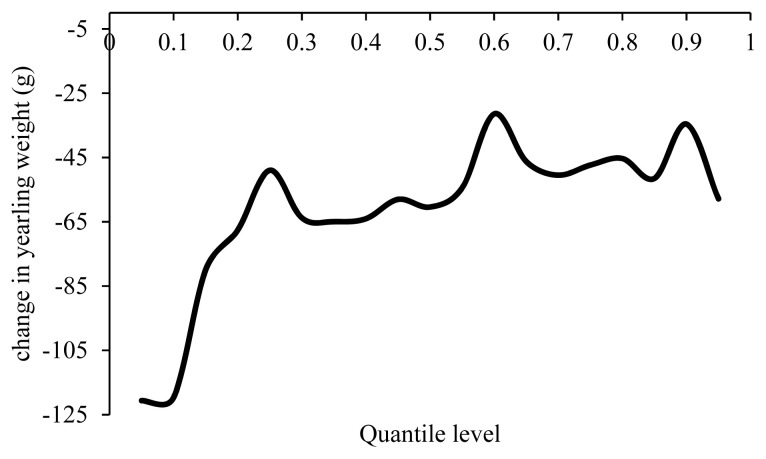
Estimated effect of inbreeding on different quantiles of yearling weight.

According to Figure 10, with an increase in the inbreeding coefficient by 1%, YW decreased 31 to 120 g, and the effect of inbreeding depression was more significant in weights below the average. The effect of inbreeding on YW was estimated −131 g in the Moghani breed and −159 g in the Baluchi breed, which were higher than the estimates of the present study [2, 13]. Yavarifard et al [8] reported a 98 g increase in YW with a 1% increase in the inbreeding coefficient which was inconsistent with the present research.

**Figure 11:**
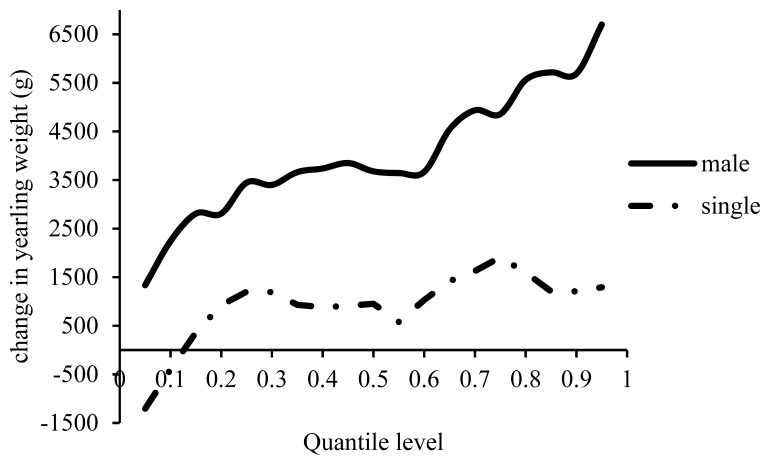
Estimated effect of inbreeding on different quantiles of yearling weight in male and single lambs.

With a 1% increase in inbreeding male lambs weighted heavier than female lambs by 1,300 to 6,600 g in all the percentiles of YW, and this difference was more significant in the higher percentiles (Figure 11). Although twins were heavier than single tones by 390 to 1,200 g at the first two quantiles, single-born lambs were generally heavier than twins at all other percentiles with a 1% increase in inbreeding. To the best of the authors’ knowledge, Ghavi Hossein-Zadeh [13] was the only researcher who compared the effect of inbreeding on YW. In Moghani sheep he obtained a decrease by 357 g in YW of male lambs, by 30 g in the females, by 183 g in the singletons and by 92 g in the twins.

Several biological and methodological variables can be used to estimate the inbreeding effect on performance traits. Negative and positive inbreeding effects are therefore usually mixed in a population [5]. The present study reported both positive and negative effects for inbreeding. In terms of age, inbreeding reduced growth traits, although its effects were dissimilar in different percentiles of growth traits. In terms of sex and birth type in each age, inbreeding differently affected the different percentiles of the growth traits; it reduced the lambs’ weight in certain percentiles, and increased in some others. Quantile regression can therefore be used to obtain more accurate estimates for the genetic parameters of growth traits. Furthermore, optimal animal weights can be predicted to reduce inbreeding depression at a given age. Quantile regression can also be useful for evaluating the effect of inbreeding on other economically important traits such as reproductive and survival performance. Further research is therefore recommended to focus on applying quantile regression models to better understand the effect of inbreeding on a wide range of traits in the Baluchi and other sheep breed.

## CONCLUSION

Quantile regression applied in this research revealed that inbreeding did not affect similarly at different percentiles of growth traits, i.e. BW, WW, 6MW, 9MW, and YW in Iranian Baluchi lambs, suggesting that at a given age and inbreeding coefficient, lambs with different sex and birth type were not equally influenced by inbreeding given that other environmental factors were also taken into account.

## Figures and Tables

**Table 1:** Descriptive statistics for the growth traits of Baluchi sheep breed

Trait		N	Mean±SE	Min	Max
Birth weight (kg)	Total	12,843	4.20±0.75	2	6.5
	Male	6,509	4.33±0.76	2	6.5
	Female	6,333	4.07±0.71	2	6.1
	Single	7,235	4.54±0.66	2	6.5
	Twin	5,607	3.77±0.63	2	6
Weaning weight (kg)	Total	11,607	21.31±5.22	10	47
	Male	5,873	22.18±5.52	10	48
	Female	5,734	20.41±4.73	10	43
	Single	6,568	23.03±4.94	10	47
	Twin	5,039	19.06±4.70	10	45
Six-month weight (kg)	Total	9,113	30.50±6.00	15	53
	Male	4,590	32.07±6.21	15	53
	Female	4,523	28.91±5.32	15	52
	Single	5,426	31.99±5.86	15	53
	Twin	3,687	28.31±5.49	15	50
Nine-month weight (kg)	Total	8,238	33.59±6.09	20	57
	Male	4,114	35.08±6.54	20	57
	Female	4,124	32.09±5.30	20	50
	Single	4,973	34.64±6.11	20	57
	Twin	3,265	31.98±5.70	20	57
	Total	7,677	38.49±7.47	20	69
Yearling weight (kg)	Male	3,750	41.06±7.79	20	69
	Female	3,927	36.03±6.21	20	60
	Single	4,623	39.36±7.50	20	66
	Twin	3,054	37.15±7.22	20	69

**Table 2:** Summary of pedigree structure for Baluchi sheep breed

Item	Number	% in total	Mean inbreeding coefficient
Total animals	13,633	100	0.0163
Inbred animals	6,047	44.35	0.0368
Non-inbred animals	7,586	55.64	0
Sires	308	2.25	-
Dams	3,747	27.48	-
Animals with both known parents	12,281	90.08	-
Animals with both unknown parents (founders)	791	5.80	-
Animals with progeny	4,055	29.74	-
Animals with no progeny	9,578	70.25	-

**Table 3:** Distribution of Baluchi sheep breed in different inbreeding classes

Class of F	Number	Frequency (%)
F = 0	7,586	55.64
0<F≤0.1	5,610	41.15
0.1<F≤0.2	359	2.63
F>0.2	78	0.58

F, inbreeding coefficient.
